# A Modularity-Based Method Reveals Mixed Modules from Chemical-Gene Heterogeneous Network

**DOI:** 10.1371/journal.pone.0125585

**Published:** 2015-04-30

**Authors:** Jianglong Song, Shihuan Tang, Xi Liu, Yibo Gao, Hongjun Yang, Peng Lu

**Affiliations:** 1 Institute of Automation, Chinese Academy of Sciences, Beijing, China; 2 Institute of Chinese Materia Medica, China Academy of Chinese Medical Sciences, Beijing, China; Texas A&M University, UNITED STATES

## Abstract

For a multicomponent therapy, molecular network is essential to uncover its specific mode of action from a holistic perspective. The molecular system of a Traditional Chinese Medicine (TCM) formula can be represented by a 2-class heterogeneous network (2-HN), which typically includes chemical similarities, chemical-target interactions and gene interactions. An important premise of uncovering the molecular mechanism is to identify mixed modules from complex chemical-gene heterogeneous network of a TCM formula. We thus proposed a novel method (MixMod) based on mixed modularity to detect accurate mixed modules from 2-HNs. At first, we compared MixMod with Clauset-Newman-Moore algorithm (CNM), Markov Cluster algorithm (MCL), Infomap and Louvain on benchmark 2-HNs with known module structure. Results showed that MixMod was superior to other methods when 2-HNs had promiscuous module structure. Then these methods were tested on a real drug-target network, in which 88 disease clusters were regarded as real modules. MixMod could identify the most accurate mixed modules from the drug-target 2-HN (normalized mutual information 0.62 and classification accuracy 0.4524). In the end, MixMod was applied to the 2-HN of Buchang naoxintong capsule (BNC) and detected 49 mixed modules. By using enrichment analysis, we investigated five mixed modules that contained primary constituents of BNC intestinal absorption liquid. As a matter of fact, the findings of *in vitro* experiments using BNC intestinal absorption liquid were found to highly accord with previous analysis. Therefore, MixMod is an effective method to detect accurate mixed modules from chemical-gene heterogeneous networks and further uncover the molecular mechanism of multicomponent therapies, especially TCM formulae.

## Introduction

Since network pharmacology emerged, multicomponent therapy becomes an important aspect of network medicine [1, 2]. A fundamental characteristic of multicomponent therapy that differs from conventional drugs is that different components of a therapy usually function in a synergistic manner to relieve complex diseases [3, 4]. Thus, TCM (Traditional Chinese Medicine) recipes containing hundreds of herbal constituents and mediating thousands of potential targets are essentially multicomponent therapies with empirical efficacy for diverse chronic disorders [4, 5]. However, the increasing amount of constituents and associated targets leads to unprecedented complexity in the pharmacology research and drug design of multicomponent therapies [6]. In fact, the molecular mechanism of most TCM formulae stay unclear [4]. Therefore, to uncover the mode of action of a classic therapy especially TCM formula is a difficult and urgent task to facilitate the development of multicomponent drug discovery.

**Fig 1:**
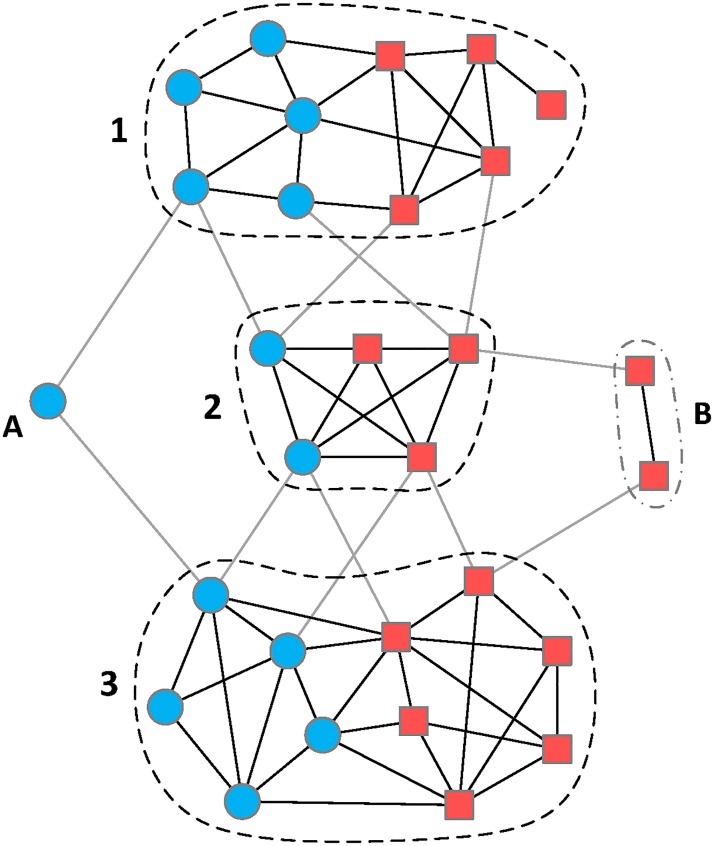
An illustration of a chemical-gene heterogeneous network. The blue nodes are chemical constituents and the red nodes represent potential gene targets. This network is an instance of 2-class heterogeneous network [9], which is more than a simple chemical-gene bipartite graph by including additional interactions between chemicals and between genes. Obviously, there are three mixed modules (1, 2, and 3) in this heterogeneous network. Each mixed module is a highly-interconnected unit in which chemicals directly or indirectly regulate the expression of corresponding genes. Additionally, module A and B are also considered as special cases of mixed module. Such modules may influence the final partition of module detection methods, but make little contribution to uncovering particular molecular mechanism.

Complex network is an essential and practical tool to model and analyze the molecular system of a multicomponent therapy [7, 8]. For example, a TCM formula usually contains hundreds of chemical constituents and these chemicals may regulate thousands of gene targets. Thus the molecular system of this TCM formula could be modeled by a 2-class heterogeneous network (2-HN) in which chemicals and gene targets are nodes and interactions between nodes are considered as links [9]. 2-HN is a kind of graph whose nodes belong to two classes and whose links connect nodes from arbitrary class. However, when the number of nodes in a network reaches high order of magnitude, it costs a lot of efforts to perform qualitative analysis from a holistic perspective. An available solution is to capture the dominant modules within a network using module detection techniques and then analyze these modules with biological knowledge [10–12]. This is based on an assumption that chemicals with similar structures usually share similar functions, namely act on same group of genes. In general, complex processes in a biological system are accomplished by the concerted action of different groups of genes [13–15]. In the chemical-gene heterogeneous network of a multicomponent therapy, the dominant modules typically contain both chemicals and gene targets (Fig 1). Such modules are called mixed modules since they contain nodes of two classes (one class is chemical and the other is gene). A mixed module indicates that the chemicals within it achieve certain functions by synergistically regulating the expression of corresponding targets [11]. Therefore, by using module detection methods, we could identify primary mixed modules from a chemical-gene molecular system and further reveal the mode of action underlying each module.

A number of methods have been proposed to detect modules from complex biological networks. Markov Cluster algorithm (MCL) is one of the earliest methods to identify highly interconnected modules from a network, which was based on random walks in the network [16]. Then a well-known hierarchical clustering method named Girvan-Newman algorithm was proposed [17]. It employed edge betweenness as a metric to decompose a network into modules. Newman and Girvan subsequently devised a quality function, commonly known as modularity, to evaluate the “goodness” of network partition (a partition of a network is the set of detected modules from the network) [18]. A number of modularity optimization methods had been developed to identify significant modules [19]. One of the most popular modularity-based methods is Louvain method with high efficiency in detecting modules in large networks [20]. In addition, Infomap, a method derived from information theory, was proposed to reveal the community structure by compressing the description of information flows in a network [21]. Although many classical methods can fulfill the task of module detection, few are particularly devised for 2-HN and certain important information is omitted by these methods in identifying mixed modules from 2-HNs (Fig 1). Therefore, a novel method is necessary in order to discover accurate mixed modules from complex chemical-gene heterogeneous networks.

Here we presented a modified measure combined with Louvain strategy to detect mixed modules from chemical-gene 2-HNs. Our method was compared with other four methods in computer-generated networks and in a real-world network. We next applied our method to the chemical-gene 2-HN of Buchang naoxintong capsule (BNC) to uncover its molecular mechanism. In vitro experiments were conducted to verify the predictions of our method. As a consequence, the computational predictions were found to agree with the findings of in vitro experiments.

## Materials and Methods

### Detect mixed module

We used 2-class heterogeneous network (2-HN) to model complex chemical-gene systems or other systems with two classes of nodes [9]. A 2-HN, as shown in Fig 1, contains two classes of nodes and three kinds of links. Thus, a 2-HN can be denoted by *G* = (*V*, *E*), with *V* = {*V*_A_, *V*_B_} and *E* = {*E*_AA_, *E*_AB_, *E*_BB_}, where *V*_A_ is the set of nodes of class *A*; *V*_B_ is node set of class *B*; *E*_AA_ is the set of links connecting *A* nodes; *E*_BB_ is the link set between *B* nodes; *E*_AB_ is the link set between *A* nodes and *B* nodes. Obviously, it can be divided into three subnetworks in terms of link category. *G* = *G*_A_ ∪ *G*_Π_ ∪ *G*_B_, where *G*_A_ = (*V*_A_, *E*_AA_) is the subnetwork of class *A*, *G*_B_ = (*V*_B_, *E*_BB_) is the subnetwork of class *B*, and *G*_Π_ = (*V*_A_, *V*_B_, *E*_AB_) is a bipartite graph connecting nodes of class *A* and class *B*. In practice, three subnetworks are usually constructed in different ways and then integrated into a 2-HN. Namely, the connectiveness, density, and even range of link weights are not homogeneous in an integrated 2-HN, but particularly consistent in any of its three subnetworks. This is the primary difference of a 2-HN from classic networks. Although classic module detection algorithms are applicable to identify mixed modules from a 2-HN, it is still necessary to devise a novel method which takes into account the heterogeneity underlying a 2-HN.

Due to the heterogeneity of 2-HN, we proposed a novel measure to evaluate the “goodness” of a partition of a 2-HN. This measure is named mixed modularity, to distinguish from classic modularity. The idea underlying mixed modularity is that, similar to 2-HN, we virtually divide a mixed module into three submodules in terms of link category, then evaluate the significance of each submodule in corresponding subnetwork, and finally sum up degrees of significance of three submodules as a unified metric for the mixed module. The mixed modularity of a partition is the summation of metrics over all mixed modules in it. Note that a module comprised of single-group nodes (module A and B in Fig 1) is also considered as a special case of mixed module, which influences the optimization of mixed modularity. In fact, mixed modularity is a linear combination of Newman-Girvan modularity for simple graph [18] and Barber modularity for bipartite graph [22]. Therefore, the mixed modularity *mQ* can be expressed as follows.

$$\begin{array}{c} mQ=\frac{1}{3}\sum_{c=1}^{n_{c}}\{[\frac{l_{Ac}}{m_{A}}-{\left(\frac{d_{Ac}}{2m_{A}}\right)}^{2}]+[\frac{l_{\Pi c}}{m_{\Pi }}-\frac{k_{\Pi c}d_{\Pi c}}{m_{\Pi }^{2}}]+[\frac{l_{Bc}}{m_{B}}-{\left(\frac{d_{Bc}}{2m_{B}}\right)}^{2}]\} \end{array}$$ (1)

where *n*_*c*_ is the number of modules in a given partition; module *c* is virtually divided into three submodules, *c* = (A*c*)∪(Π*c*)∪(B*c*), A*c* is the submodule with links from *E*_AA_, Π*c* is from *E*_AB_ and B*c* is from *E*_BB_; *l*_A*c*_ is the number of links in submodule A*c*, *m*_A_ is the size of subnetwork *G*_A_, *d*_A*c*_ is the sum of degrees of all A*c* nodes in *G*_A_; *k*_Π*c*_ is the sum of degrees of *A* nodes of Π*c* in subnetwork *G*_Π_, *d*_Π*c*_ is the sum of degrees of *B* nodes of Π*c* in *G*_Π*c*_. According to (Eq 1), mixed modularity takes into account the significance of mixed modules in three subnetworks respectively. In this way, it can partly avoid the cross-impact of different links. For example, the degree of a given node is computed separately by counting the number of links connecting nodes of same class or different classes. Besides, mixed modularity can be easily extended to a weighted version.

Using mixed modularity as the optimization function, we adopted the Louvain strategy to search for the best partition of a 2-HN [20]. We referred to this method as MixMod for convenience. The process of MixMod consists of two main phases, elaborated in S1 File. Due to Louvain strategy, this method can fast detect mixed modules from a complex 2-HN.

### Computer-generated 2-HN

Our method was compared with other four methods, including Markov Cluster algorithm (MCL) [16], Clauset-Newman-Moore algorithm (CNM) [23], Infomap [21] and Louvain method [20]. We generated 2-HNs with known module label as benchmarks to evaluate the performance of five methods. Since conventional benchmark methods like Girvan-Newman benchmark and LFR benchmark only generate simple network whose nodes belong to single class [17, 24], we hence proposed a novel method to construct benchmark 2-HNs. The procedure of 2-HN benchmark generator is presented as follows.

1. According to LFR benchmark [24], generate subnetwork *G*_A_ with preset parameters including number of nodes *N*_A_, average degree *k*_A_, maximum degree max*k*_A_ and mixing parameter *μ*_A_. The output network has *n*_A_ modules;
2. Generate subnetwork *G*_B_ as step (1), the parameters of *G*_B_ are *N*_B_, *k*_B_, max*k*_B_, *μ*_B_. The number of modules is *n*_B_;
3. Set the number of mixed modules *n*_*c*_ = min{*n*_A_, *n*_B_}, then randomly assign *A* modules and *B* modules into *n*_*c*_ clusters and make sure each cluster, namely mixed module, contains modules from both subnetworks;
4. Following the procedure of bipartite benchmark [25], place links between *A* nodes and *B* nodes in same mixed modules with probability *p* and place links between arbitrary *A* nodes and *B* nodes with probability 1 − *p*. The resulting bipartite *G*_Π_ has *n*_*c*_ modules;
5. Output the 2-HN by integrating *G*_A_, *G*_B_ and *G*_Π_, in which each node is assigned a mixed module label;

According to the procedure above, benchmark 2-HNs can be generated for testing the capability of different module detection methods. In addition, two acknowledged measures are adopted to evaluate the accuracies of modules detected by five methods, which are normalized mutual information (NMI) [26] and classification accuracy (CA) [19, 27]. Based on information theory, NMI estimates the similarity between “real” partition and “found” partition. Large NMI indicates a good partition similar to the “real” one (S1 File). Different from NMI, CA firstly determines a module matching by finding the largest overlap between pairs of modules from “real” and “found” partitions and then outputs the fraction of common nodes according to the best matching (S1 File). Large CA implies a “good” partition comprised of modules similar to the “real” one.

### Real drug-target system

Besides computer-generated benchmarks, our method was also compared with others in a real drug-target system with known module structure. Here, we regarded disease clusters as real modules in a drug-target heterogeneous network. Each disease cluster consists of drugs and genes related to that disease. This is due to an assumption that the disease clusters in a drug-target network is consistent with its module structure to some extent. Namely, drugs and genes for specific diseases may highly interact with each other and form mixed modules in the whole network. Thus, we constructed a 2-HN as a benchmark, in which all drugs and genes were assigned to disease clusters. A dataset was firstly downloaded, which contained FDA-approved drugs treating diverse diseases and disease genes curated from OMIM database [28]. From the dataset, numbers of diseases were excluded which share too many associated drugs with other diseases. As a consequence, drugs and genes for 88 diseases were curated. These drugs and genes were labeled by their associated diseases. Additional drug targets were collected from DrugBank database [29]. Each target was assigned to the disease cluster that its associated drugs belong to. The similarities between drugs were calculated using the Tanimoto coefficient of fingerprints of any two drugs through OpenBabel toolkit [30]. The Tanimoto coefficient of two fingerprints is defined as the number of common bits divided by the number of nonzero bits in either fingerprint [9, 30]. Only drug pairs with similarities equal to or larger than 0.7 were selected [31]. The interactions between all genes including drug targets and disease genes were extracted from HPRD [32], BioGRID [33], and IntAct database [34]. Each gene interaction is weighted by the number of databases including it. Finally, the benchmark 2-HN was constructed by integrating drug pairs, drug-target interactions and gene interactions, which has 88 mixed modules for diverse diseases. The measures to evaluate the performance of different methods are NMI and CA.

### Application on Buchang naoxintong capsule

We employed our method to investigate the molecular mechanism of Buchang naoxintong capsule (BNC) and then verified the predicted results via in vitro experiments. The primary task was to construct a 2-HN representing the molecular system of BNC. The chemical constituents within BNC herbs were retrieved from Chemistry Database of Chinese Academy of Sciences (http://www.organchem.csdb.cn/). Then potential targets of curated herbal constituents were extracted from CTD database [35]. The interactions between gene targets were extracted from HPRD [32], BioGrid [33], and IntAct database [34]. Similar pairs of chemical constituents were selected by calculating the Tanimoto coefficient of chemical fingerprints. The threshold for Tanimoto similarity was set to 0.7. When the 2-HN of BNC was constructed, our method was applied to the network and then essential mixed modules were detected. These identified mixed modules were further investigated through enrichment analysis using genes of each module. That is, in a given mixed module, chemical constituents function synergistically to regulate biological pathways enriched in its gene set.

### In vitro experiment and Ethics statement

We created a in vitro model to uncover the mode of action of BNC on H9c2 rat cardiomyoblasts. Adult male Sprague-Dawley rats weighing 250–270g were obtained from the Experimental Animal Center of Peking University Health Science Center, Beijing, China (Certificate NO. SCXK (Jing) 2009-0017). All animals were housed individually at 22±2°*C* with a relative humidity of 50±10% and a 12-h light/12-h dark cycle. The animals had free access to food and water. The experimental procedures were approved by the China Academy of Chinese Medical Science’s Administrative Panel on Laboratory Animal Care. All animal experiments were performed in accordance with institutional guidelines and ethics. The experiment was approved by Experimental Animal Ethics/Administration Committee of Beijing. We firstly prepared BNC intestinal absorption liquid and identify primary bioactive constituents through liquid chromatography. Then the in vitro model of hydrogen peroxide (*H*_2_ *O*_2_)-induced H9c2 rat cardiomyoblasts was created to investigate the cardioprotective effects of BNC. We conducted numerous tests on H9c2 cells, including the detection of intracellular reactive oxygen species and apoptosis, measurement of cytosolic *Ca* ^2+^ concentration and mitochondrial membrane potential. Analyzing the experiment results, a schematic map was drawn to explain the potential mechanism of BNC protecting H9c2 cardiomyoblasts [36]. Computational predictions were also compared to the results of experimental analysis, to support the availability of MixMod.

## Results

### Computer-generated benchmark

According to the procedure of benchmark 2-HN, we generated many heterogeneous networks to test the capability of our method in detecting mixed modules. For comparison, four classic methods, MCL, CNM, Infomap and Louvain, were also employed to identify mixed modules from 2-HNs. Different from conventional networks, three subnetworks of a 2-HN usually had diverse topological properties, including number of nodes, number of links, scale of link weights, network density, average degree, clustering coefficient and so on. Namely, heterogeneity could be clearly observed in 2-HNs. As a consequence, the parameters of subnetwork *G*_A_ and *G*_B_ were specified quite differently when generating benchmark 2-HNs. We primarily set *N*_A_ = 400, *k*_A_ = 4, max*k*_A_ = 16 and *N*_B_ = 600, *k*_B_ = 12, max*k*_B_ = 48. Other parameters including *μ*_A_, *μ*_B_ and *p* were carefully studied, which determined the module structure of benchmark 2-HNs. In brief, benchmark generator with small *μ*_A_, small *μ*_B_ and large *p* would result in 2-HNs with clear module structure; otherwise, the modules would not be topologically significant in the output 2-HNs.

**Fig 2:**
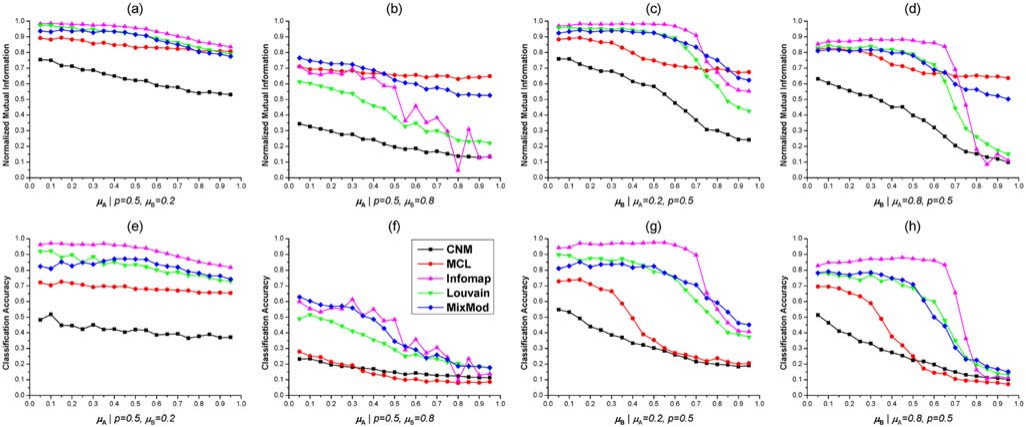
Tests of five methods on benchmark 2-HNs with varying *μ*_A_ and *μ*_B_. (**a**). Normalized Mutual Informations (NMIs) of five methods on benchmarks with *p* = 0.5 and *μ*_B_ = 0.2. (**b**). NMIs when *p* = 0.5 and *μ*_B_ = 0.8. (**c**). NMIs when *μ*_A_ = 0.2 and *p* = 0.5. (**d**). NMIs when *μ*_A_ = 0.8 and *p* = 0.5. (**e**)(**f**)(**g**)(**h**). CAs of five methods on 2-HNs with different parameters. In these figures, the variation curve of each method is marked by a unique color as shown in (**f**).

**Fig 3:**
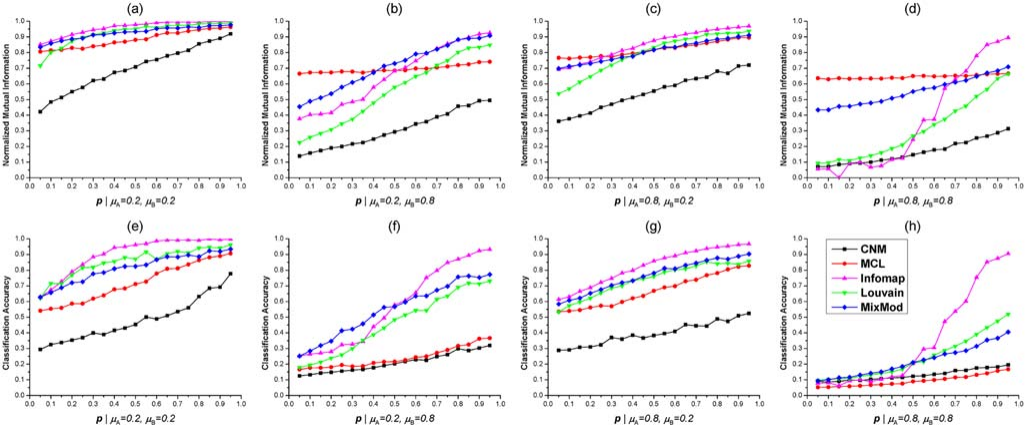
Tests of five methods on benchmark 2-HNs with fixed *μ*_A_, *μ*_B_ and varying *p*. (**a**). Normalized Mutual Informations (NMIs) of five methods on benchmarks with *μ*_A_ = 0.2 and *μ*_B_ = 0.2. (**b**). NMIs when *μ*_A_ = 0.2 and *μ*_B_ = 0.8. (**c**). NMIs when *μ*_A_ = 0.8 and *μ*_B_ = 0.2. (**d**). NMIs when *μ*_A_ = 0.8 and *μ*_B_ = 0.8. (**e**)(**f**)(**g**)(**h**). CAs of five methods on 2-HNs with different parameters. In these figures, the variation curve of each method is marked by a unique color as shown in (**h**).

With predefined parameters, we tested five methods on benchmark 2-HNs with various *μ*_A_, *μ*_B_ and *p*. To avoid contingency, the performance of five module detection methods were evaluated on 10 2-HNs with a set of same parameters. Namely, each point in Figs 2 and 3 represented the average of normalized mutual informations (NMIs) or classification accuracies (CAs) on 10 benchmark 2-HNs of same *μ*_A_, *μ*_B_ and *p*. We firstly studied the performance of five methods in detecting modules from 2-HNs with varying parameter *μ*_A_. When *p* = 0.5 and *μ*_B_ = 0.2, Infomap outperformed other methods in terms of NMI and CA (Fig 2a and 2e), and our method MixMod and Louvain method had comparable performance with NMIs around 0.9 and CAs around 0.8 for different *μ*_A_, MCL and CNM were the worst (Fig 2a and 2e). If the module stucture of subnetwork *G*_B_ was not clear (i.e., *μ*_B_ = 0.8), MixMod was the best method to identify mixed modules underlying complex 2-HNs (Fig 2b and 2f). Although MCL had larger NMIs than MixMod for *μ*_A_ ≥ 0.45, the CAs of MCL were remarkably lower than MixMod for all *μ*_A_. The abnormal phenomenon that MCL had fairly large NMIs and greatly small CAs was also observed in other situations (Fig 2). The potential reason was that MCL detected many small-sized modules and these small-sized modules resulted in a relatively large NMI (S1 Table). Besides, the NMI and CA curves of Infomap did not descend smoothly when *μ*_A_, *μ*_B_ are large and *p* is small (Figs 2 and 3). This was probably due to two reasons: first, it is not enough to perform Infomap on just 10 2-HNs with a set of fixed parameters; second, Infomap was sensitive to the network structure and not consistent when the 2-HNs had “bad” module structure (S2 Table).

We next investigated the capability of five methods in detecting mixed modules with respect to *μ*_B_. When *μ*_B_ ≤ 0.7, Infomap was superior to other methods according to both NMI and CA (Fig 2c, 2d, 2g and 2h). However, Infomap was not good as MixMod if *μ*_B_ exceeded 0.8. MixMod and Louvain method had similar NMIs or CAs when *μ*_B_ ≤ 0.6, but MixMod outperformed Louvain method for *μ*_B_ ≥ 0.7 according to NMI (Fig 2c and 2d). MCL and CNM were the worst in all situations in terms of CA. In addition, as shown in Fig 2d and 2h, the NMIs and CAs of Infomap descreased dramatically around *μ*_B_ = 0.75 and both curves reached local maximum when *μ*_B_ = 0.9. Similar phenomenon was also observed in Fig 2b and 2f when *μ*_B_ = 0.8. Moreover, all five methods achieved relatively larger NMIs or CAs when *μ*_A_ = 0.8 than when *μ*_B_ = 0.8, comparing Fig 2b and 2d, 2f and 2h. It indicated that mixed modules were difficult to detect if subnetwork *G*_B_ had insignificant module structure. The underlying reason may be the larger number of nodes in subnetwork *G*_B_ than *G*_A_ (*N*_B_ = 600, *N*_A_ = 400).

All five methods were also tested on benchmark 2-HNs with different *p*. Unlike *μ*_A_ and *μ*_B_, the NMIs and CAs were generally growing with *p* increased (Fig 3). When *p* for bipartite subnetwork *G*_Π_ was fairly large, Infomap was the best method to identify accurate mixed modules (Fig 3). CNM was the worst in most situations with various *p* (Fig 3). The NMIs for MCL were relatively large and constant, but the CAs were surprisingly small when *μ*_B_ = 0.8 (Fig 3b, 3d, 3f, and 3h). Such abnormal phenomenon had been observed and discussed above. MixMod and Louvain method had comparable performance if *μ*_B_ = 0.2 and *p* was large. When *p* was small, MixMod was slightly better than Louvain method (Fig 3a, 3c, 3e, and 3g). If *μ*_A_, *μ*_B_ were both set to 0.8 and *p* was small, MixMod exhibited the best performance among five methods (Fig 3d and 3h). Additionally, the NMI and CA curves of Infomap achieved the local minimum at *p* = 0.15 according to Fig 3d and 3h. Such unusual phenomenon was also observed when the benchmark 2-HNs had large *μ*_A_ and *μ*_B_ (Fig 2).

By testing five module detection methods on undirected, unweighted benchmark 2-HNs, we could draw several conclusions based on the comparing results. Infomap outperformed other methods when *μ*_A_, *μ*_B_ were small and *p* was large. However, the NMI or CA curves of Infomap were not smoothly changing when *μ*_A_, *μ*_B_ were large and *p* was small (Figs 2 and 3). CNM was the worst in most situations. MCL usually achieved relatively large NMIs and small CAs. MixMod and Louvain method exhibited similar performance when *μ*_A_, *μ*_B_ were small and *p* was large. According to NMI and CA, MixMod was the best method to detect mixed modules from 2-HNs with complex module structure (i.e., large *μ*_A_, *μ*_B_ and small *p*).

**Fig 4:**
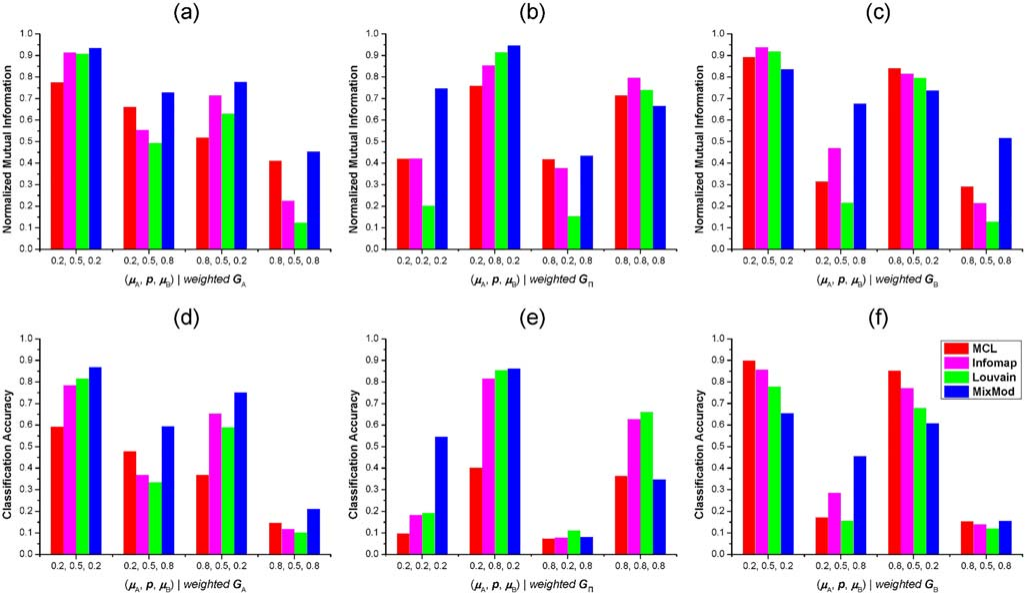
Tests of four methods on weighted benchmarks. (**a**). Normalized Mutual Informations (NMIs) of four methods on 2-HNs with different *μ*_A_, *μ*_B_ and *p*. The subnetwork *G*_A_ of each 2-HN is weighted according to the weighting scheme of LFR benchmark. (**b**). NMIs of four methods on 2-HNs with weighted subnetwork *G*_Π_. (**c**). NMIs of four methods on 2-HNs with weighted *G*_B_.

We also conducted test on undirected, weighted benchmarks using five methods. Since three subnetworks of a 2-HN were usually not constructed in a same or similar manner, we weighted one subnetwork each time to generate weighted 2-HNs. The weighting scheme used in the benchmark generator was the same as the weighted LFR benchmark [37]. Two parameters *β* and *μ*_*w*_ should be determined in the first place. Thus, *β* was set to 2 and *μ*_*w*_ equaled to *μ*_A_, 1 − *p* or *μ*_B_ for different weighted 2-HNs. Since the implementation of CNM was unable to deal with link weights, we excluded CNM in this test. Similarly, we averaged the NMIs and CAs of each method on 10 2-HNs with a set of same parameters. As shown in Fig 4, MixMod was superior to other methods in most cases, especially when *μ*_A_ and *μ*_B_ were large, and *p* was small. In the case of *μ*_A_ = 0.8, *p* = 0.8, *μ*_B_ = 0.8 for *G*_Π_ weighting benchmarks, MixMod was surprisingly worse than the other three methods (Fig 4b and 4e). Same results were also observed on *G*_B_ weighting 2-HNs with *μ*_B_ = 0.2. The situation was partly in accord with that of unweighted tests (Fig 3a and 3e). Louvain method was basicaly the worst when *μ*_B_ = 0.8 of *G*_A_ weighting, *p* = 0.2 of *G*_Π_ weighting, and *μ*_B_ = 0.8 of *G*_B_ weighting benchmarks. MCL exhibited bad performance in *G*_A_ weighting 2-HNs with *μ*_B_ = 0.2, but outperformed other methods in *G*_B_ weighting benchmarks with *μ*_B_ = 0.2. In general, Infomap was a medium method in all cases. To sum up, MixMod was a fairly good method to identify mixed modules from weighted 2-HNs.

### Drug-target heterogeneous network

Besides the artificial networks, we then compared five methods on real drug-target heterogeneous network. This network was actually a 2-HN, which consists of 277 drugs and 616 genes. There were also 82 similar drug pairs, 1234 drug-target interactions and 1253 gene interactions in the network. All drugs and genes were categorized into 88 clusters in terms of their associated diseases. A minority of genes may have multiple module labels due to diverse associations with different diseases. From the drug-target 2-HN, we selected 277 drugs and their targets to perform correlation test (S1 File). Statistical analysis indicated that there were more interactions between targets of two drugs if they had more similar structures (Spearman *ρ* = 0.117, p-value < 2.2e-16). It demonstrated that chemicals with similar structures usually act on same group of genes, which highly interact with each other. According to this fact, we subsequently compared five module detection methods using the drug-target 2-HN.

Five different methods were then applied to the drug-target 2-HN in order to identify mixed modules corresponding to disease clusters. The results of different methods were presented in Table 1. MCL and MixMod detected far more modules than other methods; while the numbers of modules detected by CNM and Louvain were much smaller than the real number of disease clusters. All methods identified modules comprised of nodes from single class (Table 1). Regardless of single-class modules, we found that MixMod detected 97 mixed modules, which was close to the number of diseases. On the other hand, MixMod was also superior to other methods, with NMI of 0.62 and CA of 0.4524 (Table 1). The low NMIs or CAs of CNM and Louvain may be due to less numbers of detected modules than the real. Namely, CNM and Louvain tended to identify modules of large size, which was consistent with the fact that Newman-Girvan modularity has resolution limit [38]. We additionally exhibited ten largest mixed modules detected by MixMod in S1 Fig. These modules were topolgically significant as shown in S1 Fig. and most of drugs and targets in same mixed module tended to associate with same disease. To sum up, MixMod was the best method to discover mixed modules from the drug-target 2-HN.

**Table 1:** Performance of five methods on real drug-target heterogeneous network.

	all modules	mixed modules	NMI	CA
CNM	35	29	0.4478	0.3852
MCL	158	111	0.6207	0.4267
Infomap	77	59	0.5337	0.4177
Louvain	25	23	0.4246	0.3516
MixMod	146	97	0.62	0.4524

All modules include mixed modules and modules of single-class nodes.

We also tested the assumption adopted in this test, which was that the disease clusters correlate with module structure of the drug-target 2-HN. Random test was employed to evaluate the significance of this hypothesis (S1 File). Statistical results basically supported this hypothesis (S1 File). Thus, we could approximately consider disease clusters as the real modules of drug-target heterogeneous network.

### Application on BNC molecular network

One great application of 2-HN and MixMod method was to study Traditional Chinese Medicine (TCM) pharmacology [4]. Here we used MixMod to identify mixed modules from the molecular network of Buchang naoxintong capsule (BNC). We collected 289 non-redundant constituents for 16 herbs in BNC from Chemistry Database (S4 Table). Then potential targets for these herbal constituents were curated from CTD database. 981 gene targets associated with 95 constituents were extracted. Using OpenBabel toolkit, fingerprint-based structure similarities between 95 chemical constituents were calculated to select similar pairs of chemicals. Gene interactions were extracted from HPRD, BioGRID and IntAct database. Each gene interaction was weighted by the number of databases including it. Finally, a complete 2-HN was constructed to model the molecular system of BNC. The BNC 2-HN contained 95 chemicals and 981 genes, with 53 chemical similarities, 1718 chemical-gene interactions and 3612 gene interactions connecting them. Before detecting mixed modules, we collected 95 chemicals and their gene targets from the 2-HN of BNC to perform correlation test (S1 File). According to statistical analysis, we found that chemicals with similar structures usually act on same group of genes (Spearman *ρ* = 0.136, p-value < 2.2e-16). Based on this fact, MixMod was applied to the 2-HN and 55 modules were detected, including 6 single-class modules. After eliminating 6 single-class modules, we performed enrichment analysis for the remaining 49 mixed modules. Note that mixed modules with genes less than 10 would be omitted in enrichment analysis. Therefore, the pharmacological functions underlying BNC could be generally uncovered according to the analysis.

We further generated a in vitro model to reveal the molecular mechanism of BNC and test the results predicted by MixMod together with enrichment analysis. Eight constituents were identified in BNC intestinal absorption liquid through liquid chromatography, which were paeoniflorin, protocatechualdehyde, salvianolic acid B, tanshinone I, caffeic acid, ferulic acid, rosmarinic acid, and hydroxysafflor yellow A. Mixed modules including these eight chemicals were particularly selected and analyzed (S6 Table). These modules were topologically significant with strong intra-module links as shown in S2 Fig. Through GO enrichment analysis, we could see that each mixed module achieved specific functions by regulating same or similar pathways (Table 2). For example, module M1 primarily participated in the bioprocess of anti-apoptosis (p-value 2.21e-11) and may also involved in the pathway of phosphorylation (p-value 3.59e-6). Tanshinone I in module M2 mediated biological processes of oxidative phosphorylation (p-value 0.004746) and oxidation reduction (p-value 0.006155). Chemicals in module M3 mainly regulated pathways related to reactive oxygen spacies (p-value 8.31e-5). Module M4 including salvianolic acid B acted on several pathways concerning apoptosis (p-value 1.42e-14). Paeoniflorin in module M5 may help to regulate the mitochondrial membrane permeability (p-value 1.54e-14). Mixed modules including rosmarinic acid and hydroxysafflor yellow A were also informative to uncover the pharmacological effect of BNC, but difficult to study using enrichment analysis due to small numbers of associated genes (S6 Table). On the other hand, in vitro experiments using BNC intestinal absorption liquid showed that BNC could protect H9c2 cardiomyocytes by enhancing antioxidative ability, activating ERK1/2 signaling pathways, inhibiting signal transduction pathways related to apoptosis and increasing mitochondrial membrane potential [36]. From the analysis above, we could conclude that our predictions by MixMod together with enrichment analysis highly accord with the experimental results. Moreover, bioactive constituents achieving specific functions were also found in mixed modules of BNC molecular network. Therefore, MixMod was an effective method to detect accurate mixed modules from chemical-gene heterogeneous networks and further uncover the molecular mechanism of TCM formulae.

**Table 2:** Enrichment analysis on essential mixed modules from the 2-HN of BNC.

Mixed Module	Enriched GO Term	P-value
M1 (**protocatechualdehyde**)	GO:0006916 anti-apoptosis	2.21E-11
	GO:0042981 regulation of apoptosis	7.61E-11
	GO:0043067 regulation of programmed cell death	8.89E-11
	GO:0010941 regulation of cell death	9.42E-11
	GO:0043066 negative regulation of apoptosis	4.67E-10
	GO:0043069 negative regulation of programmed cell death	5.49E-10
	GO:0060548 negative regulation of cell death	5.66E-10
	GO:0018105 peptidyl-serine phosphorylation	1.80E-6
	GO:0016310 phosphorylation	3.59E-6
	GO:0006468 protein amino acid phosphorylation	4.06E-6
M2 (**tanshinone I**)	GO:0006119 oxidative phosphorylation	0.004746
	GO:0055114 oxidation reduction	0.006155
M3 (**caffeic acid**, **ferulic acid**)	GO:0006749 glutathione metabolic process	1.56E-6
	GO:0006518 peptide metabolic process	2.10E-5
	GO:0000302 response to reactive oxygen species	8.31E-5
	GO:0034614 cellular response to reactive oxygen species	0.000107
	GO:0055114 oxidation reduction	0.000121
M4 (**salvianolic acid B**)	GO:0006915 apoptosis	1.42E-14
	GO:0012501 programmed cell death	1.69E-14
	GO:0008219 cell death	1.02E-13
	GO:0016265 death	1.10E-13
	GO:0042981 regulation of apoptosis	3.08E-11
M5 (**paeoniflorin**)	GO:0046902 regulation of mitochondrial membrane permeability	1.54E-14
	GO:0001836 release of cytochrome c from mitochondria	6.77E-13
	GO:0008637 apoptotic mitochondrial changes	5.64E-12
	GO:0007006 mitochondrial membrane organization	6.68E-12
	GO:0042981 regulation of apoptosis	8.87E-12

The enrichment analysis was conducted using DAVID tool [39]. Enriched terms with p-values greater than 0.01 were discarded.

## Discussion

We propose a novel measure, named mixed modularity, specified for 2-class heterogeneous networks in order to detect accurate mixed modules. Combined with the search strategy as Louvain method, our method MixMod could fast identify mixed modules from chemical-gene system modeled by a 2-HN. We firstly compare MixMod with other four methods on benchmark 2-HNs with known module structure. Results show that MixMod outperforms others when 2-HNs have promiscuous module structure (namely, large *μ*_A_, *μ*_B_ and small *p*). Then these methods are tested on a drug-target heterogeneous network with disease clusters as real modules. MixMod can identify the most accurate mixed modules from the drug-target heterogeneous network (NMI 0.62 and CA 0.4524). In the end, MixMod is applied to the 2-HN of Buchang naoxintong capsule (BNC). By using enrichment analysis, we investigate five mixed modules that include primary constituents of BNC intestinal absorption liquid. In fact, the findings of in vitro experiments are found to highly accord with previous predictions. Therefore, MixMod is an effective method to detect accurate mixed modules from chemical-gene heterogeneous networks and further uncover the molecular mechanism of multicomponent therapies, especially TCM formulae.

Compared to conventional methods, MixMod has several advantages. First, MixMod considers a 2-HN as a united combination of three subnetworks and computes the mixed modularity independently from three subnetworks. That is, topological properties concerning chemicals or genes are separately calculated in MixMod and mixed modularity. Second, according to mixed modularity, MixMod is partially robust to the structure of 2-HNs. For example, if we double the link weights of any of the three subnetworks for a given 2-HN, MixMod will identify the same partition as the original. Although MixMod is a good method to detect mixed modules, there are some disadvantages that limit the application of MixMod. Obviously, MixMod can only be applied to complex systems modeled by 2-HN. Thus MixMod is unavailable for common networks like PPI networks and drug-target bipartite networks. Even so, MixMod is still helpful and insightful because it can deal with different interactions in an integrated 2-HN. Since chemical similarities, chemical-target interactions and gene interactions are all included in an integrated network, mixed modules detected by MixMod may be quite accurate and informative to uncover molecular mechanism underlying TCM formulae.

## Supporting Information

S1 File MixMod method and additional test. (PDF)

S1 Fig Ten largest mixed modules detected by MixMod from the drug-target 2-HN. The diamond nodes are drugs and ellipse nodes are targets. Ten largest mixed modules are particularly marked by circles. Drugs and genes belonging to ten disease clusters are colored accordingly. Other nodes are all grey if they are not related to those diseases.
(TIFF)

S2 Fig Mixed modules detected by MixMod from the 2-HN of BNC. This figure presents a module network according to the final partition of the BNC 2-HN, as introduced in S1 File. A diamond node is a submodule of chemicals and an ellipse node is a submodule of genes. A mixed module is represented by two adjacent nodes (one diamond and one ellipse) of this network. A self-loop represents all interactions between nodes of a submodule. Intra-module links are colored by black and inter-module links are grey.
(TIFF)

S1 Table The number of detected modules by MCL on benchmark 2-HNs. (XLS)

S2 Table The variation of NMIs and CAs by Infomap on benchmark 2-HNs. (XLS)

S3 Table Topological properties of the drug-target heterogeneous network. (XLS)

S4 Table Herbal composition of Buchang naoxintong capsule. (XLS)

S5 Table Topological properties of the chemical-gene heterogeneous network of BNC. (XLS)

S6 Table Mixed modules including 8 bioactive constituents of BNC intestinal absorption liquid. (XLS)

S7 Table Supplementary data of real drug-target heterogeneous network and molecular network of Buchang naoxintong capsule (BNC). (a). FDA-approved drugs of drug-target heterogeneous network. (b). Drug targets of drug-target network. (c). 88 disease clusters of drug-target network. (d). Molecular network of Buchang naoxintong capsule (BNC).
(XLS)

## References

[1] Hopkins AL. Network pharmacology: the next paradigm in drug discovery. Nat Chem Biol. 2008 11;4(11):682–690. 10.1038/nchembio.118 18936753

[2] Keith CT, Borisy AA, Stockwell BR. Multicomponent therapeutics for networked systems. Nat Rev Drug Discov. 2005 1;4(1):71–78. 10.1038/nrd1609 15688074

[3] Barabasi AL, Gulbahce N, Loscalzo J. Network medicine: a network-based approach to human disease. Nat Rev Genet. 2011 1;12(1):56–68. 10.1038/nrg2918 21164525 PMC3140052

[4] Zhao J, Jiang P, Zhang W. Molecular networks for the study of TCM Pharmacology. Briefings in Bioinformatics. 2010;11(4):417–430. 10.1093/bib/bbp063 20038567

[5] Li J, Lu C, Jiang M, Niu X, Guo H, Li L, et al Traditional Chinese Medicine-Based Network Pharmacology Could Lead to New Multicompound Drug Discovery. Evidence-Based Complementary and Alternative Medicine. 2012;2012:11. 10.1155/2012/149762 PMC3541710 23346189

[6] Kitano H. A robustness-based approach to systems-oriented drug design. Nat Rev Drug Discov. 2007;6(3):202–210. 10.1038/nrd2195 17318209

[7] Li S, Zhang B. Traditional Chinese medicine network pharmacology: theory, methodology and application. Chinese Journal of Natural Medicines. 2013;11(2):110–120. 10.3724/SP.J.1009.2013.00110 23787177

[8] Liang X, Li H, Li S. A novel network pharmacology approach to analyse traditional herbal formulae: the Liu-Wei-Di-Huang pill as a case study. Mol BioSyst. 2014;10:1014–1022. 10.1039/c3mb70507b 24492828

[9] Song J, Zhang F, Tang S, Liu X, Gao Y, Lu P, et al A Module Analysis Approach to Investigate Molecular Mechanism of TCM Formula: A Trial on Shu-feng-jie-du Formula. Evidence-Based Complementary and Alternative Medicine. 2013;2013:14 10.1155/2013/731370 PMC3860149 24376467

[10] Shi Z, Derow C, Zhang B. Co-expression module analysis reveals biological processes, genomic gain, and regulatory mechanisms associated with breast cancer progression. BMC Systems Biology. 2010;4(1):74 10.1186/1752-0509-4-74 20507583 PMC2902438

[11] Li S, Zhang B, Jiang D, Wei Y, Zhang N. Herb network construction and co-module analysis for uncovering the combination rule of traditional Chinese herbal formulae. BMC Bioinformatics. 2010;11(Suppl 11):S6 10.1186/1471-2105-11-S11-S6 21172056 PMC3024874

[12] Li H, Zhao L, Zhang B, Jiang Y, Wang X, Guo Y, et al A Network Pharmacology Approach to Determine Active Compounds and Action Mechanisms of Ge-Gen-Qin-Lian Decoction for Treatment of Type 2 Diabetes. Evidence-Based Complementary and Alternative Medicine. 2014;2014:12 10.1155/2014/495840 PMC3914348 24527048

[13] Ravasz E, Somera AL, Mongru DA, Oltvai ZN, Barabsi AL. Hierarchical Organization of Modularity in Metabolic Networks. Science. 2002 8;297:1551–1555. 10.1126/science.1073374 12202830

[14] Hartwell LH, Hopfield JJ, Leibler S, Murray AW. From molecular to modular cell biology. Nature. 1999;402:C47C52 10.1038/35011540 10591225

[15] Barabasi AL, Oltvai ZN. Network biology: understanding the cell’s functional organization. Nat Rev Genet. 2004 2;5(2):101–113. 10.1038/nrg1272 14735121

[16] van Dongen SM. Graph Clustering by Flow Simulation. University of Utrecht, The Netherlands; 2000.

[17] Girvan M, Newman MEJ. Community structure in social and biological networks. Proceedings of the National Academy of Sciences. 2002;99(12):7821–7826. 10.1073/pnas.122653799 PMC122977 12060727

[18] Newman MEJ, Girvan M. Finding and evaluating community structure in networks. Phys Rev E. 2004;69(2):026113 10.1103/PhysRevE.69.026113 14995526

[19] Fortunato S. Community detection in graphs. Physics Reports. 2010;486(3–5):75–174. 10.1016/j.physrep.2009.11.002

[20] Blondel VD, Guillaume JL, Lambiotte R, Lefebvre E. Fast unfolding of communities in large networks. Journal of Statistical Mechanics: Theory and Experiment. 2008;2008(10):P10008 10.1088/1742-5468/2008/10/P10008

[21] Rosvall M, Bergstrom CT. Maps of random walks on complex networks reveal community structure. Proceedings of the National Academy of Sciences. 2008;105(4):1118–1123. 10.1073/pnas.0706851105 PMC2234100 18216267

[22] Barber MJ. Modularity and community detection in bipartite networks. Phys Rev E. 2007;76(6):066102 10.1103/PhysRevE.76.066102 18233893

[23] Clauset A, Newman MEJ, Moore C. Finding community structure in very large networks. Phys Rev E. 2004 12;70(6):066111 10.1103/PhysRevE.70.066111 15697438

[24] Lancichinetti A, Fortunato S, Radicchi F. Benchmark graphs for testing community detection algorithms. Phys Rev E. 2008;78(4):046110 10.1103/PhysRevE.78.046110 18999496

[25] Guimer R, Sales-Pardo M, Amaral LAN. Module identification in bipartite and directed networks. Phys Rev E. 2007;76(3):036102 10.1103/PhysRevE.76.036102 PMC2262941 17930301

[26] Danon L, Diaz-Guilera A, Duch J, Arenas A. Comparing community structure identification. Journal of Statistical Mechanics: Theory and Experiment. 2005;2005(09):P09008 10.1088/1742-5468/2005/09/P09008

[27] Meila M, Heckerman D. An Experimental Comparison of Model-Based Clustering Methods. Mach Learn. 2001 1;42(1–2):9–29. 10.1023/A:1007648401407

[28] Yildirim MA, Goh KI, Cusick ME, Barabasi AL, Vidal M. Drug-target network. Nat Biotech. 2007;25(10):1119–1126. 10.1038/nbt1338 17921997

[29] Knox C, Law V, Jewison T, Liu P, Ly S, Frolkis A, et al DrugBank 3.0: a comprehensive resource for ‘Omics’ research on drugs. Nucleic Acids Research. 2011;39(suppl 1):D1035–D1041. 10.1093/nar/gkq1126 21059682 PMC3013709

[30] O’Boyle N, Banck M, James C, Morley C, Vandermeersch T, Hutchison G. Open Babel: An open chemical toolbox. Journal of Cheminformatics. 2011;3(1):33 10.1186/1758-2946-3-33 21982300 PMC3198950

[31] O’Boyle N, Morley C, Hutchison G. Pybel: a Python wrapper for the OpenBabel cheminformatics toolkit. Chemistry Central Journal. 2008;2(1):5 10.1186/1752-153X-2-5 18328109 PMC2270842

[32] Keshava Prasad TS, Goel R, Kandasamy K, Keerthikumar S, Kumar S, Mathivanan S, et al Human Protein Reference Database2009 update. Nucleic Acids Research. 2009;37(suppl 1):D767–D772. 10.1093/nar/gkn892 18988627 PMC2686490

[33] Chatr-aryamontri A, Breitkreutz BJ, Heinicke S, Boucher L, Winter A, Stark C, et al The BioGRID interaction database: 2013 update. Nucleic Acids Research. 2013;41(D1):D816–D823. 10.1093/nar/gks1158 23203989 PMC3531226

[34] Kerrien S, Aranda B, Breuza L, Bridge A, Broackes-Carter F, Chen C, et al The IntAct molecular interaction database in 2012. Nucleic Acids Research. 2012;40(D1):D841–D846. 10.1093/nar/gkr1088 22121220 PMC3245075

[35] Davis AP, Murphy CG, Johnson R, Lay JM, Lennon-Hopkins K, Saraceni-Richards C, et al The Comparative Toxicogenomics Database: update 2013. Nucleic Acids Research. 2013;41(D1):D1104–D1114. 10.1093/nar/gks994 23093600 PMC3531134

[36] Zhang F, Huang B, Zhao Y, Tang S, Xu H, Wang L, et al BNC Protects H9c2 Cardiomyoblasts from H2O2-Induced Oxidative Injury through ERK1/2 Signaling Pathway. Evidence-Based Complementary and Alternative Medicine. 2013;2013:12. 10.1155/2013/802784 PMC3810482 24223618

[37] Lancichinetti A, Fortunato S. Benchmarks for testing community detection algorithms on directed and weighted graphs with overlapping communities. Phys Rev E. 2009 7;80:016118 10.1103/PhysRevE.80.016118 19658785

[38] Fortunato S, Barthlemy M. Resolution limit in community detection. Proceedings of the National Academy of Sciences. 2007;104(1):36–41. 10.1073/pnas.0605965104 PMC1765466 17190818

[39] Huang DW, Sherman BT, Lempicki RA. Systematic and integrative analysis of large gene lists using DAVID bioinformatics resources. Nat Protocols. 2008 12;4(1):44–57. 10.1038/nprot.2008.211 19131956

