# Supplementary material for: A Modularity-Based Method Reveals Mixed Modules from Chemical-Gene Heterogeneous Network
Source: PLoS One. 2015 Apr 30;10(4):e0125585. doi: 10.1371/journal.pone.0125585 (PMC4416014; doi:10.1371/journal.pone.0125585)
Supplement: S1 File — (PDF) [file pone.0125585.s001.pdf]

Supplementary File S1

1 MixMod method

A 2-class heterogeneous network (2-HN) can be denoted by $G = (V, E)$, with $V = \{V_A, V_B\}$ and $E = \{E_{AA}, E_{AB}, E_{BB}\}$. Thus, a 2-HN could be divided into three subnetworks in terms of link category. In practice, we usually construct a 2-HN by integrating $G_A = (V_A, E_{AA})$, $G_\Pi = (V_A, V_B, V_{AB})$ and $G_B = (V_B, E_{BB})$ into a complex heterogeneous network. In order to detect reasonable mixed modules from a 2-HN, we proposed a new measure, namely mixed modularity, by integrating Newman-Girvan modularity and Barber modularity. The mixed modularity can be expressed as follows.

$$mQ = \frac{1}{3}\sum_{c=1}^{n_c}\left[\frac{l_{Ac}}{m_\mathrm{A}} - \left(\frac{d_{Ac}}{2m_\mathrm{A}}\right)^2\right] + \frac{1}{3}\sum_{c=1}^{n_c}\left[\frac{l_{\Pi c}}{m_\Pi} - \frac{k_{\Pi c} d_{\Pi c}}{m_\Pi^2}\right] + \frac{1}{3}\sum_{c=1}^{n_c}\left[\frac{l_{Bc}}{m_\mathrm{B}} - \left(\frac{d_{Bc}}{2m_\mathrm{B}}\right)^2\right] \tag{1}$$

where n_c is the number of modules in a given partition; module c is virtually separated into three submodules, $c = (\mathrm{A}c) \cup (\Pi c) \cup (\mathrm{B}c)$, A$c$ is the submodule with links from E_AA, Πc is from E_AB and Bc is from E_BB; $l_{\mathrm{A}c}$ is the number of edges in submodule Ac, m_A is the size of subnetwork G_A, $d_{\mathrm{A}c}$ is the sum of degrees of all Ac nodes in G_A; $k_{\Pi c}$ is the sum of degrees of A nodes of Πc in subnetwork G_Π, $d_{\Pi c}$ is the sum of degrees of B nodes of Πc in $G_{\Pi c}$.

With mixed modularity as the optimal function, the Louvain strategy is then adopted to search for an optimal partition. We name such a method MixMod for convenience. The procedure of MixMod method is illustrated in Figure 1. Most of MixMod is similar to the process of Louvain method [1], except a little change in the second phase. At first, each node in the 2-HN is assigned to individual modules. So, the initial partition consists of modules as many as the number of nodes. In the first phase, we calculate the gain of mixed modularity by removing node i from its module and by placing i in the module of its neighbor j. The node i is then placed in the module for which the gain is maximum and positive. If all gains are less than zero, we keep node i in its original module. Such process is performed repeatedly and sequentially for all nodes until no positive gain is achieved. In the second phase, a new network is constructed according to the partition in the first phase that reaches the local maxima of mixed modularity. If a module in previous partition includes nodes of both group A and group B, two differnt nodes are added into the new network to represent A nodes and B nodes in the module respectively. Thus these two nodes belong to a same module (Figure 1). The weights of links in the new network are given by the sum of weights of links between two sets of nodes in the original network. It is obvious that this new network is also a 2-HN with nodes from A and B group. We use this new network as input and then repeat previous two phases until the mixed modularity is maximum. In the end, mixed modules could be identified corresponding to the final partition.

2 Benchmark evaluation

One important measure to evaluate the precision of benchmark tests is normalized mutual information (NMI). It quantifies the similarity between "real" and "found" modules from a given network. The normalized mutual

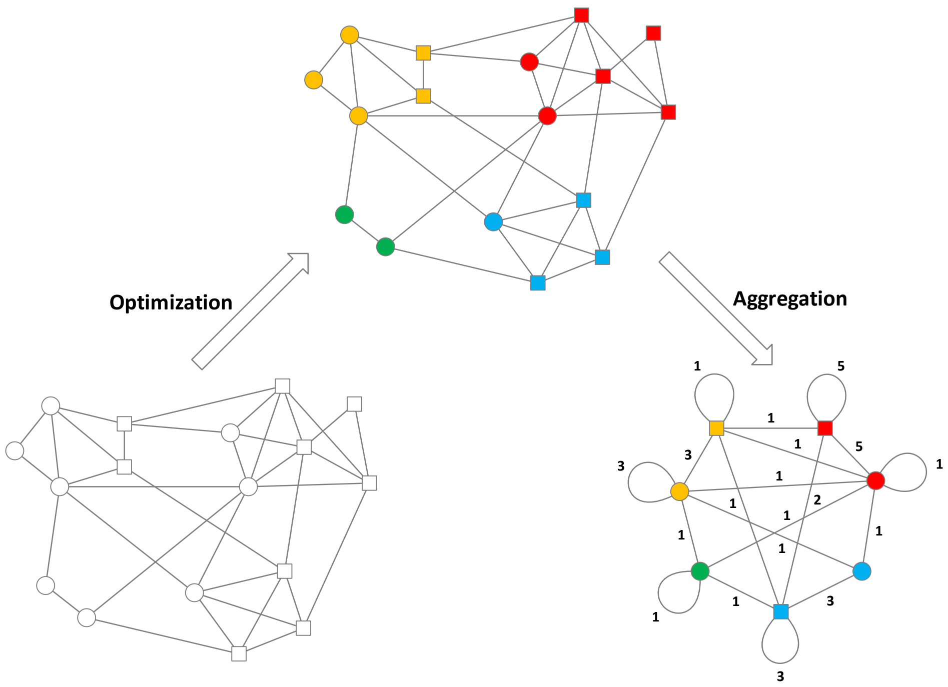

Figure 1: Main steps of Louvain optimization for MixMod method. This illustration is modified based on the original process of Louvain method [1].

information is calculated base on a confusion matrix, where the rows correspond to the "real" modules and the columns correspond to the "found" modules [2]. The NMI is defined as follows.

$$I(X,Y) = \frac{-2\sum_{i=1}^{c_X}\sum_{j=1}^{c_Y} N_{ij}\log\left(\frac{N_{ij}N}{N_{i\cdot}N_{\cdot j}}\right)}{\sum_{i=1}^{c_X} N_{i\cdot}\log\left(\frac{N_{i\cdot}}{N}\right) + \sum_{j=1}^{c_Y} N_{\cdot j}\log\left(\frac{N_{\cdot j}}{N}\right)} \tag{2}$$

where the number of "real" modules is denoted c_X and the number of "found" modules is denoted c_Y; N_{ij} is the number of common nodes between the "real" module i and the "found" module j; $N_{i\cdot}$ is the sum over row i and $N_{\cdot j}$ is the sum over column j. The NMI ranges from 0 to 1, and large NMI indicates a good partition similar to the "real" one.

Another measure to evaluate the performance of module detection methods is classification accuracy (CA). To calculate CA, we firstly find the best matching with the largest overlap between pairs of modules from "real" and "found" partition and then measure the faction of overlapping nodes over all nodes in a network [3, 4]. The classification accuracy (CA) is defined as follows.

$$R(X,Y) = \frac{1}{n}\max_{f}\sum_{k=1}^{\min\{c_X,c_Y\}} n_{kf(k)} \tag{3}$$

where n is the number of all nodes in a 2-HN; c_X and c_Y are the numbers of "real" modules and "found" modules respectively; $f(\cdot)$ is an one-to-one mapping between "real" and "found" partitions. The difficult part in calculating CA is to find the optimal matching $f(\cdot)$ with maximum overlaps. We use Kuhn-Munkres algorithm to solve this problem [5]. Thus, CA could be computed fast and quantify the similarity between detected modules and "real" ones.

3 Randomized drug-target network

Based on the drug-target network, we use a "self-contained" test to verify the hypothesis that disease clusters in the network partly correlates with its module structure. A "shuffle" process is performed on the drug-target network to generate random 2-HNs with various module structure. Three subnetworks are shuffled independently and then combined into a random 2-HN. The procedure of "shuffle" operation is as follows. For every link (u, v) in each subnetwork, we randomly choose a pair of nodes (u', v') to replace (u, v), that is, to remove link (u, v) and add link (u', v') with the same weight as (u, v). Thereinto, u' is randomly chosen from the neighbors of node v with a probability proportion to the weight of link (u', v), and so is v'. Note that the weights of reduplicate links are added together. After the drug-target 2-HN is randomized, we evaluate the correlation between disease clusters and module structure of random 2-HNs by NMI and CA, because the module structure can be represented by detected modules using different methods mentioned above. Such process is repeated for 1000 times. We define P_{NMI} as the fraction of random 2-HNs with larger NMIs than the original drug-target network with respect to disease clusters, and P_{CA} as the fraction of random 2-HNs with larger CAs than the original.

Table 1: Random test using four different methods.

	P_{NMI}	P_{CA}
MCL	$< 10^{-3}$	$< 10^{-3}$
Infomap	0.133	$< 10^{-3}$
Louvain	0.633	0.034
MixMod	$< 10^{-3}$	$< 10^{-3}$

In previous test of four methods on real drug-target system, we made an assumption that disease clusters in the drug-target 2-HN correlated with its module structure to some extent. Here, we further verified this assumption using random test. Random 2-HNs were generated by shuffling links of the real drug-target network. NMI and CA were employed to estimate the correlation between true disease clusters and the module structure of random 2-HNs, since the module structure could be represented by detected modules using different methods. The random process was repeated for 1000 times. P_{NMI} and P_{CA} were p-values for this random test. We excluded CNM in random test since the implementation of CNM was unable to deal with link weights. P_{NMI} of MCL and MixMod were smaller than 10^{-3}. However, P_{NMI} of Infomap and Louvain were not significant enough (larger than 0.05). On the other hand, P_{CA} of four methods were all less than 0.05. Thus, the p-values were generally significant for the random test. As a consequence, we can conclude that disease clusters in the drug-target network partly correlated with its module structure (Table 1).

4 Correlation test

The detection of mixed modules from chemical-gene heterogeneous network was based on an assumption that chemicals with similar structures usually act on same group of genes, between which there are plenty of interactions. To verify this assumption, we investigated the correlation between chemical similarity and the number of target interactions. The structural similarity was calculated by the Tanimoto coefficient of fingerprints of two chemicals using OpenBabel toolkit [6]. The Tanimoto coefficient was defined as follows.

$$sim(c_1, c_2) = \frac{c}{a + b - c} \tag{4}$$

where c_1 and c_2 are two chemicals; a and b are the bit lengths of c_1 and c_2 fingerprints, respectively; and c is the number of common bits between c_1 and c_2 fingerprints.

For a pair of chemicals, we then computed the number of interactions between targets of two chemicals. If two chemicals had identical targets, the interaction number was set to 2 for each identical target. For example, chemical c_1 had target TNF and c_2 also had target TNF, we specified that there were 2 interactions between TNF of c_1 and TNF of c_2. Since different chemicals always regulated different number of targets, we normalized the interaction number by dividing the geometric mean of target numbers of two chemicals. Thus, the number of interactions between target sets of chemical c_1 and c_2 was defined as follows.

$$noi(c_1, c_2) = \frac{\sum_{t_i \in T_1, t_j \in T_2} I(t_i, t_j)}{\sqrt{|T_1| \cdot |T_2|}} \tag{5}$$

where T_1 is the target set of c_1, t_i is one of its targets; $I(t_i, t_j)$ is 2 if t_i is identical to t_j, $I(t_i, t_j)$ is 1 if there is an interaction between t_i and t_j, otherwise 0; $|T_1|$ is the number of targets in set T_1. For each pair of chemicals, we could compute the structural similarity and the number of interactions between them. We performed correlation test using this data to investigate whether similar chemicals act on same group of genes.

We used two examples to verify previous assumption. From real drug-target 2-HN, 277 drugs and their targets were selected for correlation test. The interactions between targets were curated from HPRD, BioGRID, and IntAct database. Statistic analysis indicated that there were more interactions between targets of two drugs if they had more similar structures (Spearman ρ=0.117, p-value<2.2e-16). Besides, we collected 95 chemicals and their associated genes from the 2-HN of BNC for correlation test. All interactions were extracted from HPRD, BioGRID and IntAct database. We could draw a same conclusion as previously stated (Spearman ρ=0.136, p-value<2.2e-16). Therefore, the assumption that chemicals with similar structures usually act on same group of genes was correct according to statistical analysis.

5 Gene network of BNC

Although the predicted results derived from the BNC 2-HN were generally proved by in vitro experiments, we performed more tests to show the advantage of using the 2-HN of BNC. At first, the whole set of gene targets associated with chemicals of BNC were employed to discover enriched GO terms using DAVID tool [7]. Top 40 enriched entries were listed in Table 2. Most of these GO terms were related to signal pathways concerning anti-apoptosis and oxidative stress. However, in vitro experiments using BNC intestinal absorption liquid showed that BNC could protect H9c2 cardiomyocytes by enhancing antioxidative ability, activating ERK1/2 signaling pathways, inhibiting signal transduction pathways related to apoptosis and increasing mitochondrial membrane potential [8]. We found that no terms concerning mitochondrial membrane permeability were ranked in top 40 (Table 2). It implied that the prediction using all gene targets of BNC was partially accurate and important information was omitted. In fact, the term GO:0051881 (regulation of mitochondrial membrane potential) were ranked 313 in the result of enrichment analysis.

On the other hand, we used gene network of BNC to investigate potential functions of the constituents of BNC. The gene network was composed of 981 gene targets of BNC and 3612 interactions between them. Since all nodes of this gene network belonged to single class, MixMod was not applicable to this situation. Hence, we employed MCL to identify significant gene modules. As a result, 130 gene modules were detected by MCL. The DAVID tool was then employed to analyze the enriched GO terms for modules with no less than 10 genes. After enrichment analysis, we could discover the potential functions of each chemical of BNC based on the overlapping between its gene targets and detected modules. A chemical was thought to regulate a gene

Table 2: Top 40 enriched GO terms using all gene targets associated with chemicals of BNC.

Rank	Enriched GO Term	P-value
1	GO:0010033 response to organic substance	4.46E-53
2	GO:0010941 regulation of cell death	7.48E-44
3	GO:0043067 regulation of programmed cell death	1.91E-43
4	GO:0042981 regulation of apoptosis	8.74E-43
5	GO:0042127 regulation of cell proliferation	1.11E-39
6	GO:0009719 response to endogenous stimulus	6.44E-37
7	GO:0043066 negative regulation of apoptosis	6.56E-35
8	GO:0043069 negative regulation of programmed cell death	2.33E-34
9	GO:0060548 negative regulation of cell death	3.00E-34
10	GO:0042592 homeostatic process	8.40E-34
11	GO:0009725 response to hormone stimulus	9.22E-33
12	GO:0009611 response to wounding	2.15E-30
13	GO:0042493 response to drug	1.26E-28
14	GO:0009991 response to extracellular stimulus	4.32E-28
15	GO:0010035 response to inorganic substance	1.32E-27
16	GO:0006979 response to oxidative stress	1.20E-26
17	GO:0006916 anti-apoptosis	1.23E-26
18	GO:0008202 steroid metabolic process	2.42E-26
19	GO:0008284 positive regulation of cell proliferation	2.46E-26
20	GO:0048878 chemical homeostasis	3.16E-26
21	GO:0031667 response to nutrient levels	3.50E-26
22	GO:0048545 response to steroid hormone stimulus	3.44E-25
23	GO:0007610 behavior	5.83E-24
24	GO:0051094 positive regulation of developmental process	4.76E-23
25	GO:0014070 response to organic cyclic substance	1.11E-22
26	GO:0019216 regulation of lipid metabolic process	2.29E-22
27	GO:0010942 positive regulation of cell death	1.63E-20
28	GO:0019725 cellular homeostasis	3.70E-20
29	GO:0043068 positive regulation of programmed cell death	4.43E-20
30	GO:0009628 response to abiotic stimulus	5.55E-20
31	GO:0043065 positive regulation of apoptosis	1.03E-19
32	GO:0045597 positive regulation of cell differentiation	2.00E-19
33	GO:0002237 response to molecule of bacterial origin	2.02E-19
34	GO:0007242 intracellular signaling cascade	6.14E-19
35	GO:0010604 positive regulation of macromolecule metabolic process	6.46E-19
36	GO:0007584 response to nutrient	6.55E-19
37	GO:0032101 regulation of response to external stimulus	6.78E-19
38	GO:0051173 positive regulation of nitrogen compound metabolic process	8.29E-19
39	GO:0070482 response to oxygen levels	8.90E-19
40	GO:0009891 positive regulation of biosynthetic process	1.19E-18

module if most of its targets were found in the module. Thus, it may achieve certain functions by mediating the biological processes in which its targets participated. Since eight bioactive chemicals were identified in BNC intestinal absorption liquid, predicted results with regard to these chemicals were presented in Table 3. According to Table 3, we found that protocatechualdehyde primarily mediated the pathways of inflammatory response (p-value 0.00075); tanshinone I mainly participated in biological processes related to transcription and gene expression (p-value 1.62E-15); caffeic acid and ferulic acid influenced the proliferation of cells (p-value 0.002123); paeoniflorin generally regulated the processes of DNA damage and repair (p-value 5.14E-11). Gene modules associated with other bioactive chemicals were too small to analyze using enrichment analysis. It was obvious that the enriched terms greatly differed from the findings of in vitro experiments. Therefore, MixMod together with 2-HN was an appropriate method to uncover the mechanism of TCM formulae.

References

[1] Blondel VD, Guillaume JL, Lambiotte R, Lefebvre E. Fast unfolding of communities in large networks. Journal of Statistical Mechanics: Theory and Experiment. 2008;2008(10):P10008.

[2] Danon L, Diaz-Guilera A, Duch J, Arenas A. Comparing community structure identification. Journal of Statistical Mechanics: Theory and Experiment. 2005;2005(09):P09008.

[3] Meila M, Heckerman D. An Experimental Comparison of Model-Based Clustering Methods. Mach Learn. 2001 Jan;42(1-2):9–29.

[4] Fortunato S. Community detection in graphs. Physics Reports. 2010;486(3-5):75 – 174.

[5] Munkres J. Algorithms for the Assignment and Transportation Problems. Journal of the Society for Industrial and Applied Mathematics. 1957;5(1):32–38.

[6] O'Boyle N, Banck M, James C, Morley C, Vandermeersch T, Hutchison G. Open Babel: An open chemical toolbox. Journal of Cheminformatics. 2011;3(1):33.

[7] Huang DW, Sherman BT, Lempicki RA. Systematic and integrative analysis of large gene lists using DAVID bioinformatics resources. Nat Protocols. 2008 Dec;4(1):44–57.

[8] Zhang F, Huang B, Zhao Y, Tang S, Xu H, Wang L, et al. BNC Protects H9c2 Cardiomyoblasts from H2O2-Induced Oxidative Injury through ERK1/2 Signaling Pathway. Evidence-Based Complementary and Alternative Medicine. 2013;2013:12.

Table 3: Bioactive chemicals of BNC and top enriched GO terms of their regulating gene modules.

Chemical	Enriched GO Term	P-value
**protocatechualdehyde**	GO:0006953 acute-phase response	0.00011
	GO:0010033 response to organic substance	0.00021
	GO:0032101 regulation of response to external stimulus	0.00051
	GO:0050727 regulation of inflammatory response	0.00075
	GO:0006954 inflammatory response	0.00092
	GO:0007155 cell adhesion	0.00104
	GO:0022610 biological adhesion	0.00105
	GO:0009611 response to wounding	0.00134
	GO:0002526 acute inflammatory response	0.00157
	GO:0051353 positive regulation of oxidoreductase activity	0.00170
**tanshinone I**	GO:0006357 regulation of transcription from RNA polymerase II promoter	1.62E-15
	GO:0006355 regulation of transcription, DNA-dependent	3.94E-15
	GO:0030522 intracellular receptor-mediated signaling pathway	6.97E-15
	GO:0051252 regulation of RNA metabolic process	7.41E-15
	GO:0045944 positive regulation of transcription from RNA polymerase II promoter	5.15E-14
	GO:0045893 positive regulation of transcription, DNA-dependent	2.23E-13
	GO:0051254 positive regulation of RNA metabolic process	2.57E-13
	GO:0051173 positive regulation of nitrogen compound metabolic process	2.82E-13
	GO:0010628 positive regulation of gene expression	5.25E-13
	GO:0045449 regulation of transcription	6.11E-13
**caffeic acid**	GO:0008283 cell proliferation	0.002123
**ferulic acid**	GO:0022403 cell cycle phase	0.008782
**paeoniflorin**	GO:0033554 cellular response to stress	2.76E-11
	GO:0000075 cell cycle checkpoint	4.82E-11
	GO:0006974 response to DNA damage stimulus	5.14E-11
	GO:0006281 DNA repair	5.36E-11
	GO:0051726 regulation of cell cycle	2.76E-10
	GO:0042770 DNA damage response, signal transduction	9.16E-10
	GO:0006259 DNA metabolic process	1.70E-9
	GO:0000077 DNA damage checkpoint	1.80E-9
	GO:0042981 regulation of apoptosis	2.80E-9
	GO:0031570 DNA integrity checkpoint	3.00E-9
