# Supplementary figures and images for: A Modularity-Based Method Reveals Mixed Modules from Chemical-Gene Heterogeneous Network
Source: PLoS One. 2015 Apr 30;10(4):e0125585. doi: 10.1371/journal.pone.0125585 (PMC4416014; doi:10.1371/journal.pone.0125585)

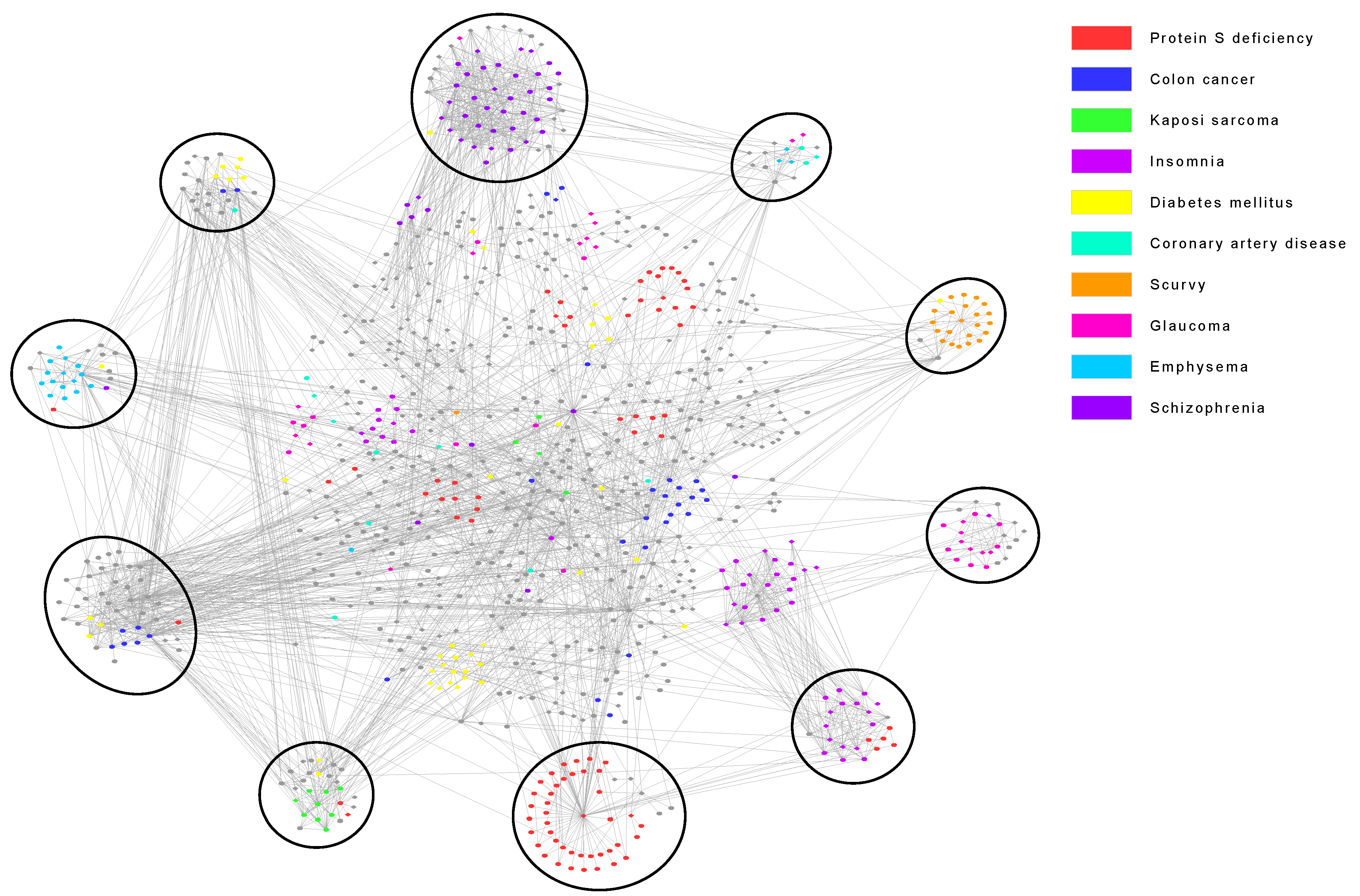

Supplement: S1 Fig — The diamond nodes are drugs and ellipse nodes are targets. Ten largest mixed modules are particularly marked by circles. Drugs and genes belonging to ten disease clusters are colored accordingly. Other nodes are all grey if they are not related to those diseases. (TIFF) [file pone.0125585.s002.tiff]

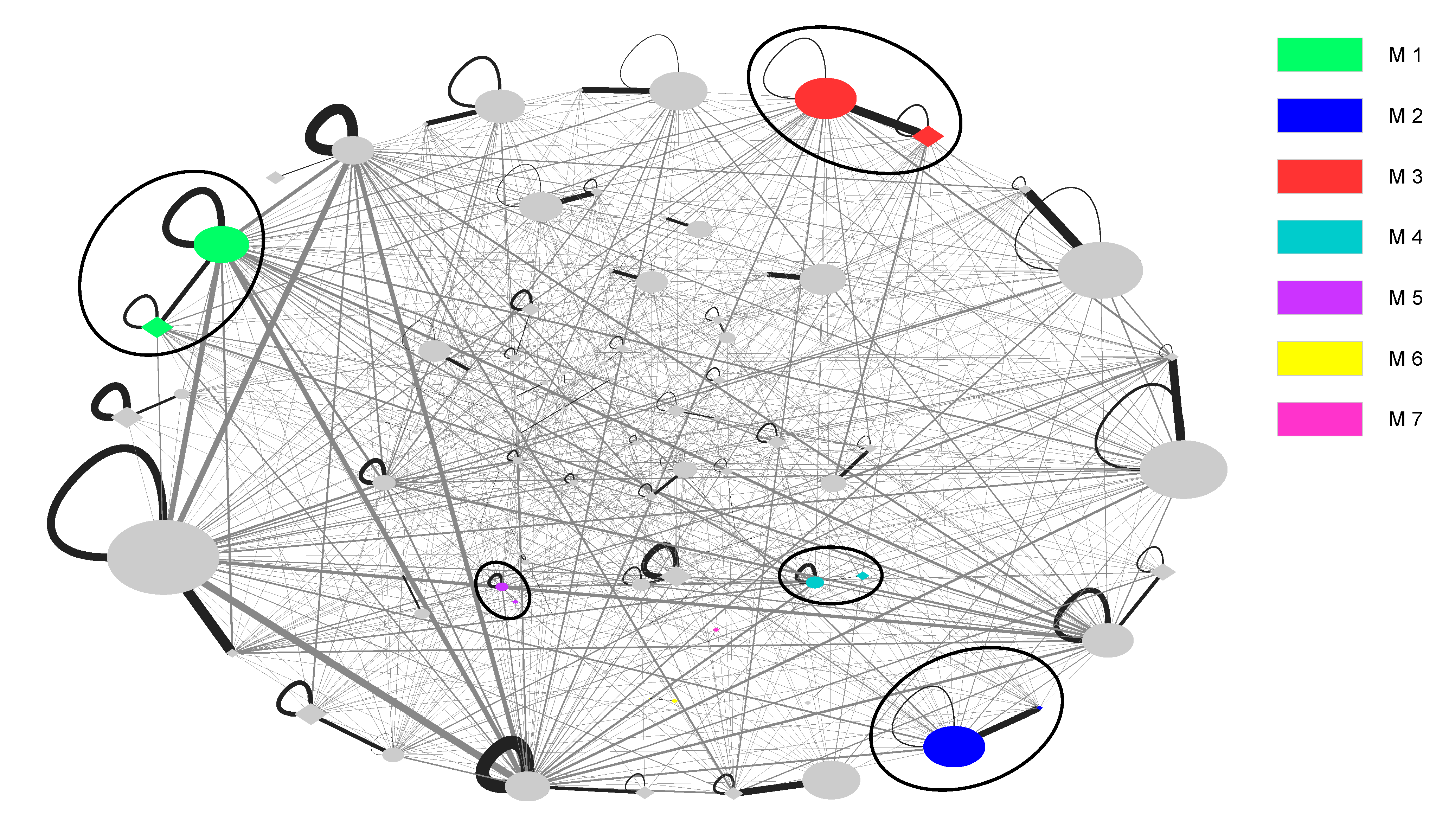

Supplement: S2 Fig — This figure presents a module network according to the final partition of the BNC 2-HN, as introduced in S1 File. A diamond node is a submodule of chemicals and an ellipse node is a submodule of genes. A mixed module is represented by two adjacent nodes (one diamond and one ellipse) of this network. A self-loop represents all interactions between nodes of a submodule. Intra-module links are colored by black and inter-module links are grey. (TIFF) [file pone.0125585.s003.tiff]
